# Reliability, validity, and simplification of the Chinese version of the Global Pain Scale in patients with rheumatoid arthritis

**DOI:** 10.1186/s12912-023-01664-4

**Published:** 2024-01-05

**Authors:** Haoyang Chen, Xiaoxiao Wang, Ting Bai, Hengmei Cui, Songsong Shi, Yunyun Li, Guang-yin Xu, Huiling Li, Biyu Shen

**Affiliations:** 1 Department of Nursing, Shanghai Children’s Medical Center, School of Medicine, Shanghai Jiao Tong University, 1678 Dongfang Road, Pudong New Area, Shanghai, China; 2 Shanghai Jiao Tong University School of Nursing, Shanghai, China; 3 Research Center of Clinical Epidemiology, Peking University Third Hospital, Beijing, China; 4 Department of Nursing, Affiliated Hospital of Xuzhou Medical University, Jiangsu, China; 5 Center for Translational Pain Medicine, Institute of Neuroscience, Soochow University, Suzhou, China; 6 Department of Nursing, Nursing School of Soochow University, 1 Shizi Street, Wuzhong District, Suzhou, China

**Keywords:** Rheumatoid arthritis, Global Pain Scale, Reliability, Validity, Short-version, Item response theory, Computerized adaptive testing.

## Abstract

**Background:**

Persistent pain is the most reported symptom in patients with rheumatoid arthritis (RA); however, effective and brief assessment tools are lacking. We validated the Chinese version of the Global Pain Scale (C-GPS) in Chinese patients with RA and proposed a short version of the C-GPS (s-C-GPS).

**Method:**

The study was conducted using a face-to-face questionnaire survey with a multicenter cross-sectional design from March to December 2019. Patients aged > 18 years who met the RA diagnostic criteria were included. Based on the classical test theory (CTT) and the item response theory (IRT), we assessed the validity and reliability of the C-GPS and the adaptability of each item. An s-C-GPS was developed using IRT-based computerized adaptive testing (CAT) analytics.

**Results:**

In total, 580 patients with RA (mean age, 51.04 ± 24.65 years; mean BMI, 22.36 ± 4.07 kg/m^2^), including 513 (88.4%) women, were included. Most participants lived in a suburb (49.3%), were employed (72.2%) and married (91.2%), reported 9–12 years of education (66.9%), and had partial medical insurance (57.8%). Approximately 88.1% smoked and 84.5% drank alcohol. Analysis of the CTT demonstrated that all items in the C-GPS were positively correlated with the total scale score, and the factor loadings of all these items were > 0.870. A significant positive relationship was found between the Visual Analog Scale (VAS) and the C-GPS. IRT analysis showed that discrimination of the C-GPS was between 2.271 and 3.312, and items 6, 8, 13, 14, and 16 provided a large amount of information. Based on the CAT and clinical practice, six items covering four dimensions were included to form the s-C-GPS, all of which had very high discrimination. The s-C-GPS positively correlated with the VAS.

**Conclusion:**

The C-GPS has good reliability and validity and can be used to evaluate pain in RA patients from a Chinese cultural background. The s-C-GPS, which contains six items, has good criterion validity and may be suitable for pain assessment in busy clinical practice.

**Trial registration:**

This cross-sectional study was registered in the Chinese Clinical Trial Registry (ChiCTR1800020343), granted on December 25, 2018.

**Supplementary Information:**

The online version contains supplementary material available at 10.1186/s12912-023-01664-4.

## Background

Pain is defined as an unpleasant sensory and emotional experience associated with or resembling actual or potential tissue damage [1]. It is cited as the most prominent symptom of many diseases [2] such as rheumatoid arthritis (RA) [3]. RA is the most common autoimmune arthritis, with a prevalence of up to 1% [4], and it can lead to periarticular bone erosion. Pain is the most common and earliest symptom in patients with RA [5]. Despite the optimal control of inflammation, persistent pain is frequently a major and common concern [6], with approximately 38.4% patients persistently experiencing moderate to severe pain [7]. Pain in RA is caused by multiple factors such as inflammation, secondary osteoarthritis, and central and peripheral sensitization [8], which can result in psychological discomfort, an increased risk of anxiety and depression, decreased physical and social functioning, and increased use of healthcare services.

The management and treatment of pain are vital clinical concerns in this population, and standardized nursing management for pain can greatly benefit patients. In the Nursing Science Precision Health Model (NSPH), symptom-based precision measurement is the primary module [9]. Unfortunately, clinical monitoring indicators for patients with RA usually cannot reflect the level of pain experienced by patients [10]. Consequently, development of an accurate and objective pain assessment tool is crucial for not only identifying the presence of pain but also evaluating the factors affecting it. Currently, the Numerical Rating Scale (NRS), Verbal Description Scale (VDS), and Visual Analog Scale (VAS) [11] are commonly used clinical and research tools for assessing pain in patients with RA. Although they are easy to use, these scales have a single dimension and cannot fully capture the multidimensional characteristics of pain in patients with RA. Therefore, a more comprehensive pain assessment tool should be adopted when considering the intricate and variable nature of the mechanisms underlying RA-related pain. Furthermore, the tool should be concise and user-friendly to accommodate the demands of a busy clinical setting. The Global Pain Scale (GPS), a multidimensional, comprehensive pain assessment tool developed by Gentile et al. in 2011 [12], comprises four dimensions, including pain, feelings, clinical outcomes, and activities. The GPS has been translated into Turkish [13], Spanish [14], and Chinese [15]; its reliability and validity have been confirmed, and it is widely used for research and clinical purposes. However, to the best of our knowledge, evidence of its application in patients with RA is lacking. Targeting and specifically assessing patients with RA can help reveal the novelty and complexity of disease-specific pain patterns, inform personalized treatment and management strategies, and improve the quality of life of patients with RA. Thus, the first aim of this study was to evaluate the validity and reliability of the Chinese version of the GPS (C-GPS) in patients with RA.

Most scales have been developed and assessed based on the classical test theory (CTT); however, brevity and clarity in operation are the major advantages. The disadvantage of the CTT is that it cannot judge the real item difficulty parameter and the participants’ ability level [16]. The Item response theory (IRT) [17], also called item characteristic curve, is a method used to explore the relationship between participants’ responses to different measurable items and their underlying latent traits. Compared with the CTT, the IRT can evaluate every participants’ ability level and measurement error through the model.

In this study, we first evaluated the validity and reliability of the C-GPS in patients with RA using the CTT; subsequently, we assessed the adaptability of each item using the IRT. The IRT allows for the development or enhancement of instruments by determining the discrimination and difficulty of items. Accordingly, the second aim of this study was to develop a short-form of the C-GPS (s-C-GPS) using IRT-based computerized adaptive testing (CAT) analytics, a system that tailors items for each respondent based on their prior answers and personability.

## Methods

### Aims

This study aimed to validate the C-GPS in Chinese patients with RA and to propose a s-C-GPS.

### Study design and participants

This multicenter, cross-sectional study was conducted using convenience sampling. Patients with RA were recruited from five hospitals between March and December 2019. The inclusion criteria were as follows: patients who met the diagnosis of RA according to the American Rheumatism Association 1987 revised criteria, those aged over 18 years, those able to interact in Chinese efficiently, and those willing to provide written informed consent. Patients with cognitive impairment or severe underlying diseases such as cancer and stroke were excluded. A total of 603 patients with RA who met the criteria were consecutively invited to participate in this study, and 580 (96.2%) were included in the analysis. The study was registered in the Chinese Clinical Trial Registry (ChiCTR1800020343), granted on December 25, 2018.

### Questionnaire

A structured questionnaire was designed and used to collect data. The questionnaire comprised four parts: sociodemographic characteristics (sex, age, body mass index [BMI], location, marital status, education, work status, insurance status, and yearly income), health status (including smoking, drinking, and history of chronic diseases [such as hypertension, diabetes, coronary heart disease, nephropathy, and cardiopulmonary disease]) and records of RA (including disease duration, disease activity, and medication status), exercise (frequency per week and duration), and pain assessment (using C-GPS and visual analog scale [VAS] scores). The C-GPS, which contains 20 items (with four dimensions: pain, feelings, clinical outcomes, and activities), is valid and reliable. The participants provided their responses on an 11-point scale (from 0 to 10). Participants on the pain subscale indicated their current level of pain, their highest, worst, and average pain levels during the preceding week, as well as whether they had experienced less pain. For pain intensity, the VAS scale is most commonly anchored by “no pain” (score of 0) and “pain as bad as it could be,” or “worst imaginable pain” (score of 100 [100-mm scale]).

### Data collection

Patients who met the inclusion criteria were enrolled after the instructions were explained to them, and informed consent was obtained. Subsequently, the participants were given a structured questionnaire containing the C-GPS and VAS. Finally, we thanked the patients for their participation in the study.

To reduce survey bias, graduate students with a background in rheumatology were selected as the investigators. Before the formal study began, we created survey manuals and trained the investigators on the study sections, methods, and caveats. Regular data sampling was performed to verify the accuracy of data entry, and all the data collected was evaluated by the researchers.

### Data analysis

Statistical analyses were performed using IBM SPSS Statistics 26.0 (Armonk, NY: IBM Corp) and NCSS 12.0 (NCSS, LLC. Kaysville, Utah, USA). Deletions and imputations were used to replace the missing data. If the number of missing items on the GPS exceeded 20%, the sample was deleted. Mean substitution and multiple imputations, based on the results of Little’s MCAR chi-square test, were performed to handle the missing values of the deleted data. Continuous variables are expressed as medians with interquartile ranges and means with standard deviations, and categorical variables are expressed as percentages. The Mann-Whitney U and Kruskal-Wallis H tests were used to examine the inter-group differences. Pearson’s correlations and single-construct factor analyses were used to evaluate the structural validity of the scale. The Keiser-Mayer-Olkin (KMO) and Bartlett sphericity tests were used to check whether the scale was appropriate for single-construct factor analysis. The level of significance was set at a *p*-value of 0.05. Based on the variables contained in the factor construct, a single-construct factor analysis was performed and one common factor was limited and extracted. Subsequently, the item with a high load was retained according to the high or low factor loadings of the measured items. Cronbach’s coefficient alpha (α) test was applied for reliability analysis. Correlation analysis was used to evaluate the structural validity of the scale between the VAS and the C-GPS.

The IRT models were estimated using the Itm package in R (v4.0.2; R Core Team 2021). The IRT-based CAT was simulated using Firestar 1.5.1. The item parameter and ability estimates were obtained using a graded response model (GRM) [18]. The abbreviated form of the C-GPS was developed using CAT analytics.

### Ethical considerations

This study was registered in the Chinese Clinical Trial Registry (ChiCTR1800020343). The Ethics Committee of the Second Affiliated Hospital of Nantong University approved on December 18 2018. Informed consent was obtained from all participants before enrollment in the study, and all procedures followed the Declaration of Helsinki and the Ethical Guidelines for Clinical Research Involving Human Subjects in China.

## Results

### Characteristics of the population

A total of 603 patients with RA were consecutively invited to participate; from these, 23 were considered ineligible (missing answers or highly repetitive answers), and 580 (96.2%) participants, including 513 (88.4%) women, were eventually included in the study. From the 580 patients, 255 (44.0%) were from Nantong, 120 (20.7%) from Henan, 101 (17.4%) from Suzhou, 55 (9.5%) from Changzhou, and 49 (8.4%) from Shanghai. The mean age was 51.04 ± 24.65 years and the mean BMI was 22.36 ± 4.07 kg/m^2^. Most participants lived in a suburb (49.3%; n = 286), were employed (72.2%; n = 419) and married (91.2%; n = 529), reported 9–12 years of education (66.9%; n = 388), and had partial medical insurance (57.8%; n = 335). Approximately 88.1% (n = 511) smoked and 84.5% (n = 490) drank alcohol. A total of 31.7% participants had a yearly per-capita income of < 15,000 RMB. The mean disease duration was 4 years. Approximately 14.7% patients had hypertension, 5% had diabetes, 6% had coronary heart disease, 3.8% had nephropathy, and 12.1% had another cardiopulmonary disease.

### Reliability and validity analysis based on the CTT

#### Pearson’s correlation analysis

The results of the correlation analysis revealed that all 20 items were positively correlated with the total GPS score, with correlation coefficients ranging between 0.829 and 0.910 (*p* < 0.001; Additional File 1).

#### Construct factor analysis

Construct factor analysis was applied to explore the structural validity of the scale, and the results of the KMO and Bartlett sphericity tests (KMO: 0.980, chi-square statistic:15967.408, Bartlett significance *p* < 0.001) indicated that the overall correlation matrix had common factors, which was perfectly appropriate for conducting the factor analysis.

Single-construct factor analysis showed (Additional Files 2–9) that all the factor loadings of all the measuring items were > 0.870, and all the sum of squares of the loadings were extracted over 80%, indicating that the four extracted factors were reasonable and the scale had good structural validity.

#### Criterion validity

The VAS is internationally recognized as the gold standard for pain evaluation. Criterion validity analysis showed a significant positive correlation between the VAS and the C-GPS (r = 0.568, *p* < 0.05), and the four domains of the C-GPS (r = 0.5, 0.55, 0.57, 0.55, all *p* < 0.05) were all positively correlated with the VAS, indicating that the scale criterion validity was good.

#### Reliability analysis

The Cronbach’s alpha coefficients for the four dimensions and 20 items of the GPS were > 0.700, indicating that the scale demonstrated good reliability (Additional Files 10–13).

### IRT analysis

#### The discrimination and difficulty level of the C-GPS

**Fig. 1:**
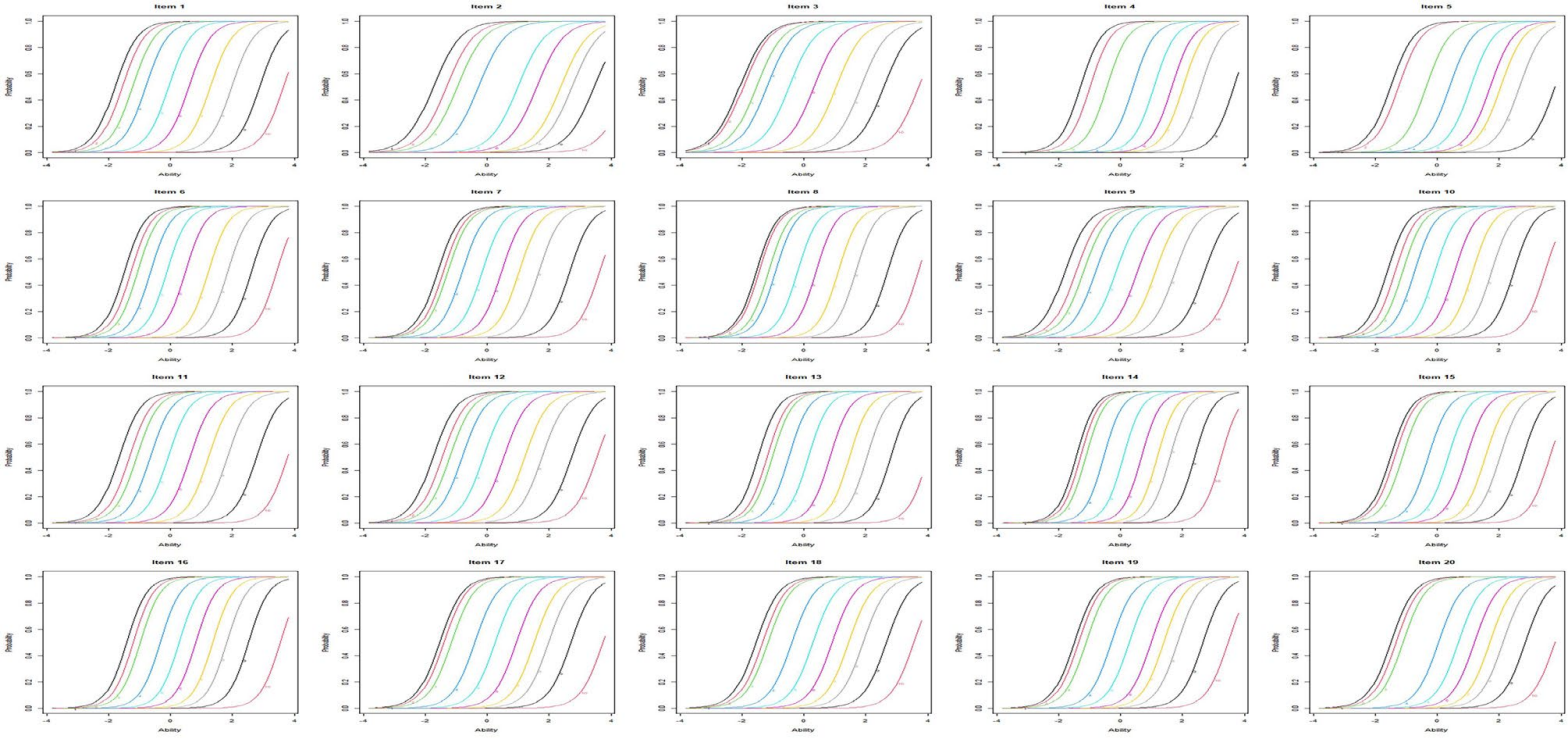
The item characteristic curves for all 20 items in the Itm package in R (v4.0.2; R Core Team 2021)

The discrimination and difficulty levels of each item estimated from the GRM model are reported in Additional File 14. The discrimination was between 2.271 (item 2) and 3.312 (item 14) (Additional File 14), suggesting high discrimination, and all items demonstrated a good ability to distinguish the presence of pain in patients with RA (Fig. 1). The difficulty level gradually increased as the difficulty parameter (abscissa) increased, indicating a greater pain severity (Fig. 1).

#### The item information function (IIF) of the C-GPS

**Fig. 2:**
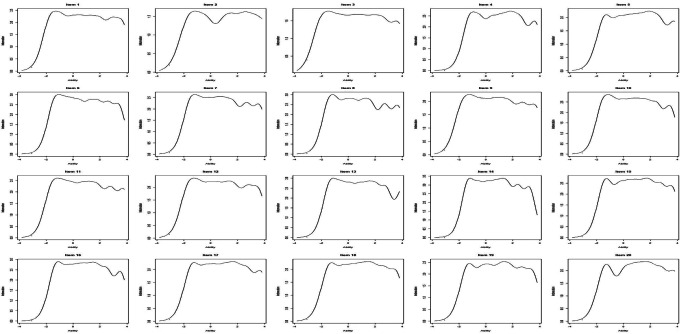
The item information function curves for all 20 items in the Itm package in R (v4.0.2; R Core Team 2021)

Considering the IIF of the C-GPS: items 6, 8, 13, 14, and 16 provided a high amount of information; items 1, 4, 7, 10, 11, 15, and 19 provided a medium amount of information; and items 2, 3, 5, 9, 12, 17, 18, and 20 provided a low amount of information. Items 4, 5, 15, 17, 18, 19, and 20 provide information on high to very high levels of pain, whereas the other items provide information on low to very low levels of pain (Fig. 2).

#### The item information and stand error function for C-GPS

**Fig. 3:**
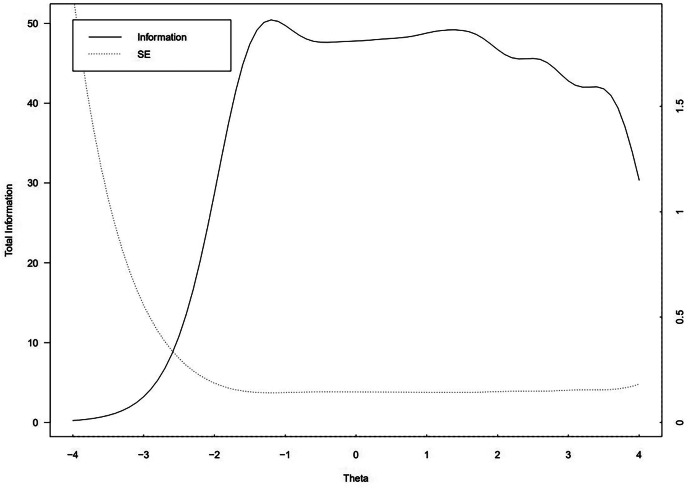
The test information curves of the Itm package in R (v4.0.2; R Core Team 2021) under the Graded Response Model (GRM). (*Note.* Latent trait θ is shown on the horizontal axis, the θ range from − 0.9 to 3.5, and the amount of information and standard error generated by testing at any trait level is shown on the vertical axis. The peak of the curve corresponds to a high reliability value at a theta value of -0.9, suggesting that C-GPS is reliable for mild to very severe chronic pain)

The TIF of the C-GPS showed an asymptotic curve with a range of reliability starting from a theta value of -0.9 to a value of 3.5, from mild to very high levels of chronic pain. The peak of the curve corresponded to the theta value of -0.9 showing a high-reliability value. Thus, the C-GPS was reliable for mild to very high levels of chronic pain but not for low levels of pain (Fig. 3).

#### Derivation of the C-GPS

Based on the parameters from the calibration test data, the difficulty and proportion selected by the CAT were used to select the items. Initially, an item was administered based on the examinee’s ability estimate. The item that provided the most information on the examinee’s assessed ability level was presented next. This process continued until the examinee’s standard error of measurement was 0.3 or a maximum of 12 items were administered. The frequency with which the items were administered in the CAT simulations was used to determine their usefulness.

Across a large sample, the IRT and the CAT of the GPS identified five items with a high discrimination ability and sufficiently variable difficulties in discriminating between individuals who reported being troubled by different levels of pain (Table 1). The selected items included items 6 (during the past week, I felt afraid), 8 (during the past week, I felt tired), 13 (during the past week, I was less independent), 14 (during the past week, I was unable to work or perform normal tasks), and 16 (during the past week, I was not able to go to the store). Using these items, a short form of the standard C-GPS consisting of five items was constructed (score range: 0–50). There was a strong positive correlation between the scores on this short form and the standard C-GPS (Spearman’s r = 0.965, *p* < 0.001). However, the short form only contained three domains: the pain domain was removed for every item that was rarely used by the CAT, and from the clinical application perspective, we included the item with the highest discrimination and highest selected proportion (item 4: During the past week, my average pain has been) in the pain domain to form the short version.

**Table 1:** Item usage and selection in the CAT for s-C-GPS (n = 580)

Domain	C-GPS item	Score Range	Usage (%)	s-C-GPS (0–60)
Pain	My current pain is	0–10	0.9	
Pain	During the past week, the best my pain has been	0–10	0	
Pain	During the past week, the worst my pain has been	0–10	0	
Pain	During the past week, my average pain has been	0–10	1.4	Y
Pain	During the past 3 months, my average pain has been	0–10	0	
	During the past week, I have felt			
Feelings	Afraid	0–10	70.9	Y
Feelings	Depressed	0–10	0.5	
Feelings	Tired	0–10	75.3	Y
Feelings	Anxious	0–10	0	
Feelings	Stressed	0–10	4.3	
	During the past week,			
Clinical outcomes	I had trouble sleeping	0–10	0.5	
Clinical outcomes	I had trouble feeling comfortable	0–10	0.2	
Clinical outcomes	I was less independent	0–10	88.1	Y
Clinical outcomes	I was unable to work (or perform normal tasks)	0–10	100	Y
Clinical outcomes	I needed to take more medication	0–10	12.4	
	During the past week, I was not able to			
Activities	Go to the store	0–10	69.8	Y
Activities	Do chores in my home	0–10	0	
Activities	Enjoy my friends and family	0–10	0	
Activities	Exercise (including walking)	0–10	0.2	
Activities	Participate in my favorite hobbies	0–10	0	

Note: CAT, Computerized Adaptive Test; GPS, Global Pain Scale; s-C-GPS, short forms of the standard Chinese version of GPS; Y, Yes

Finally, an s-C-GPS containing six items (four dimensions) with very high discrimination (all items’ discrimination parameter values were over 1.70) was constructed (score range: 0–60) (Table 2). The results of the content validity analysis showed a significant positive correlation between the s-C-GPS and the VAS (r = 0.570, *p* < 0.05), and the r-value was slightly higher than that between the GPS and VAS (r = 0.568), indicating that the scale criterion validity was good.

**Table 2:** The items and discrimination of the s-C-GPS

Domain	s-C-GPS item	Discrimination
Pain	During the past week, my average pain has been	2.949
Feelings	During the past week, I have felt: Afraid	3.07
	During the past week, I have felt: Tired	3.078
Clinical outcomes	During the past week, I was less independent	3.086
	During the past week, I was unable to work (or perform normal tasks)	3.312
Activities	During the past week, I was not able to Go to the store	3.046

Note: GPS, Global Pain Scale; s-C-GPS, short forms of the standard Chinese version of GPS

## Discussion

RA is a common autoimmune disease. Its high incidence and chronicity make it an important health concern worldwide. Compared with other pain conditions, RA often results in multisystem involvement that affects the patients’ quality of life and health status. Its characteristics include symmetrical joint involvement, systemic symptoms, and involvement of autoimmune mechanisms, making it different from other pain-related diseases. RA often leads to severe functional impairment and disability, placing a significant burden on patients and society. Therefore, more attention should be paid to RA and measures should be taken to improve its prevention, treatment, and management in order to improve the quality of life of patients and reduce its burden on society.

In this multi-center cross-sectional study, under the framework of the CTT and the IRT, we first evaluated the validity and reliability of the C-GPS in patients with RA and the adaptability of each item. IRT-based CAT analyses were then conducted to construct the s-C-GPS. Overall, the results demonstrated the good validity and reliability of the C-GPS in patients with RA, with high discrimination and sufficiently variable difficulties. In addition, the s-C-GPS containing six items (four domains) was proposed, all of which had very high discrimination and higher content validity with the VAS than with the C-GPS.

The International Association for the Study of Pain defines pain as “an unpleasant sensory and emotional experience associated with or resembling that associated with actual or potential tissue damage” [19]. The experience of pain is highly variable and influenced by a variety of factors, including physical, psychological, social, and cultural factors. Analgesics and nonsteroidal anti-inflammatory drugs are widely used to control pain [20]. The multiple mechanisms underlying RA-related pain [21] may involve inflammation, central sensitization [22, 23], joint damage, and mental and psychological factors. Because pain is subjective and multifaceted, we can provide a more comprehensive assessment of pain symptoms from physical, mental, functional, and other aspects, making it more “precise,” only by increasing the multidimensionality and comprehensiveness of pain measurement tools. Under the concept of NSPH, the assessment of a patient’s pain is the foundation of pain management, not only through pain recognition but also through assessment of the efficacy of analgesics, which should be administered dynamically and with timely feedback. A pain assessment tool should be multidimensional and accurate so that it can improve the status of patients and thus have a positive effect on treatment. Pain relief is one of the treatment goals for patients with RA. Thus, quantitative pain assessment may enable evaluation of the effectiveness of treatment and management strategies, providing a basis for clinical decision-making.

Originally, the GPS was developed to measure a patients’ chronic pain experiences by Douglas et al. in 2011 [12]. It is a comprehensive evaluation of pain with the advantages of being concise and easily interpreted. Hence, the GPS is used in a wide range of clinical settings to assess various sorts of pain [24–26]. However, no studies have validated the reliability of the GPS in patients with RA. The basic principle of the CTT is to regard the observed score as a linear combination of the underlying true score and random error [27]. The CTT is simple and easy to master and is mainly used to evaluate reliability and validity. The results of the CTT show that the C-GPS has high construct validity, criterion validity, and internal reliability. More importantly, the C-GPS consists of 20 questions across four dimensions: pain, feelings, clinical outcomes, and activities, which can accurately reflect the pain level of patients with RA, and the dimensions do not overlap. However, item difficulty calculated using the CTT depends on both the item content and the participant’s level of ability. Thus, for any given item with a high score, the CTT cannot determine whether the subject’s ability level is extremely high, or whether the test is too easy. It is worth noting that the item parameters based on the CTT varied significantly across different samples, limiting the utility of these statistics. The IRT solves these problems by fitting a model that estimates the probability of a correct answer depending on the examinee’s ability level (latent variable) and the characteristics of each item [28]. The IRT provides item discriminatory and difficulty characteristics that are independent of the study sample and helps identify redundant items. In addition, IRT models can estimate measurement errors at each level of the ability scale and are particularly useful when the focus is on improving individual items or targeting specific ability ranges.

The IRT analyses showed that the discrimination of each item was between 2.271 and 3.312, suggesting that all items demonstrated good ability to distinguish the presence of pain in patients with RA. In this multilevel scoring system, the difficulty values are strictly monotonically increasing, with higher scores indicating greater difficulty and pain severity. In the present study, the IRT analyses revealed results that were not obtained using the CTT. The C-GPS provided the largest amount of information for individuals with low to very high levels of pain, indicating the unique advantages of the C-GPS in targeting patients with moderate to severe levels of chronic pain.

The IRT identifies redundant items and helps create a short version of the GPS. Originally, the short version contained five items extracted based on the CAT, covering three different domains of pain. As pain is highly prevalent in patients with RA, these participants had similar scores on the pain dimension; that is, they had similar degrees of contribution for each item. Therefore, items of the pain dimension cannot be selected to form a brief scale. In clinical practice, the item on the pain dimension is reasonable and necessary in a pain assessment scale; therefore, we included the item with the highest discrimination in the pain domain back in the short form. Hence, six items covering four dimensions were included in the s-C-GPS. The s-C-GPS strongly correlated with C-GPS and had a scale criterion validity similar to that of C-GPS. These results suggest that the s-C-GPS is reliable and may serve as an alternative for the assessment of chronic pain in patients with RA when the original C-GPS cannot be feasibly administered in busy clinical practice.

### Limitations

This study has some limitations. Similar to the development of many abbreviated test versions, the s-C-GPS was derived from the administration of the standard version. Hence, the short form has not been administered as a unique test, and the total administration time has not been determined yet. Furthermore, the diagnostic accuracy of s-C-GPS requires further validation.

## Conclusion

This is the first study to validate the GPS for pain assessment in patients with RA. The IRT elicited additional information regarding the validity of the examinations. Second, we developed a s-C-GPS using more rigorous methods, that is, using the IRT and the CAT, in a large, broad sample, which may be more suitable for busy clinical practice.

### Electronic supplementary material

Below is the link to the electronic supplementary material.

Supplementary Material 1

## Data Availability

The datasets used and analyzed in the current study are available from the corresponding author upon reasonable request.

## List of abbreviations

RA: rheumatoid arthritis
NSPH: Nursing Science Precision Health Model
VAS: visual analog scale
VDS: verbal description scale
NRS: numeric rating scale
GPS: Global Pain Scale
C-GPS: Chinese version of the Global Pain Scale
CTT: classical test theory
IRT: item response theory
s-C-GPS: short version of the Chinese version of the Global Pain Scale
CAT: computerized adaptive testing
KMO: Keiser-Mayer-Olkin
GRM: graded response model
