# Supplementary material for: Reliability, validity, and simplification of the Chinese version of the Global Pain Scale in patients with rheumatoid arthritis
Source: BMC Nurs. 2024 Jan 5;23:20. doi: 10.1186/s12912-023-01664-4 (PMC10768464; doi:10.1186/s12912-023-01664-4)
Supplement: Supplementary file 1 — Supplementary Material 1 [file 12912_2023_1664_MOESM1_ESM.docx]

**Supplementary appendix**

**supplement to: Reliability, validity, and simplification of the Chinese version of the Global Pain Scale in patients with rheumatoid arthritis**

**Additional File 1: Item analysis for the C-GPS**

| **Secondary-level indexes** | **Item** | **Pearson correlation (R) coefficients between GPS total score** | ***P* value** |
| --- | --- | --- | --- |
| Pain | 1 | 0.845 | <0.001 |
|  | 2 | 0.846 | <0.001 |
|  | 3 | 0.829 | <0.001 |
|  | 4 | 0.896 | <0.001 |
|  | 5 | 0.873 | <0.001 |
| Feelings | 6 | 0.890 | <0.001 |
|  | 7 | 0.878 | <0.001 |
|  | 8 | 0.883 | <0.001 |
|  | 9 | 0.864 | <0.001 |
|  | 10 | 0.893 | <0.001 |
| Clinical outcomes | 11 | 0.878 | <0.001 |
|  | 12 | 0.872 | <0.001 |
|  | 13 | 0.910 | <0.001 |
|  | 14 | 0.910 | <0.001 |
|  | 15 | 0.901 | <0.001 |
| Activities | 16 | 0.903 | <0.001 |
|  | 17 | 0.879 | <0.001 |
|  | 18 | 0.878 | <0.001 |
|  | 19 | 0.872 | <0.001 |
|  | 20 | 0.889 | <0.001 |

**Additional File 1: The item analysis for the C-GPS.** Results from the correlation analysis reveal that all 20 items positively correlated with GPS total score, with correlation coefficients ranging between 0.829~0.910, *P*<0.001.

**Additional File 2: Factor analysis of Pain with number of components and total variance explained**

| **Component** | **Initial eigenvalues** | | | **Extraction sums of squared loadings** | | |
| --- | --- | --- | --- | --- | --- | --- |
|  | **Total** | **% of Variance** | **Cumulative %** | **Total** | **% of Variance** | **Cumulative %** |
| 1 | 4.152 | 83.044 | 83.044 | 4.152 | 83.044 | 83.044 |
| 2 | 0.364 | 7.280 | 90.324 | .. | .. | .. |
| 3 | 0.235 | 4.693 | 95.017 | .. | .. | .. |
| 4 | 0.136 | 2.717 | 97.734 | .. | .. | .. |
| 5 | 0.113 | 2.266 | 100.000 | .. | .. | .. |

**Additional File 3: Component matrix of Pain**

| **Item** | **Component 1** |
| --- | --- |
| 1 | 0.909 |
| 2 | 0.918 |
| 3 | 0.874 |
| 4 | 0.945 |
| 5 | 0.909 |

**Additional File 2: Factor analysis of Pain with number of components and total variance explained and Additional File 3: Factor analysis of Pain with number of components and total variance explained.** The sum of squares, loading of factor extracted in Pain factor construct, was 83.044% (Supplemental Table 2), and factor loadings of all measuring items above 0.870.

**Additional File 4: Factor analysis of Feelings with number of components and total variance explained**

| **Component** | **Initial eigenvalues** | | | **Extraction sums of squared loadings** | | |
| --- | --- | --- | --- | --- | --- | --- |
|  | **Total** | **% of Variance** | **Cumulative %** | **Total** | **% of Variance** | **Cumulative %** |
| 1 | 4.264 | 85.282 | 85.282 | 4.264 | 85.282 | 85.282 |
| 2 | 0.233 | 4.666 | 89.948 | .. | .. | .. |
| 3 | 0.79 | 3.587 | 93.535 | .. | .. | .. |
| 4 | 0.70 | 3.396 | 96.932 | .. | .. | .. |
| 5 | 0.53 | 3.068 | 100.000 | .. | .. | .. |

**Additional File 5: Component matrix of Feelings**

| **Item** | **Component 1** |
| --- | --- |
| 6 | 0.926 |
| 7 | 0.926 |
| 8 | 0.932 |
| 9 | 0.905 |
| 10 | 0.927 |

**Additional File 4 Factor analysis of Pain with number of components and total variance explained and Additional File 5 Component matrix of Feelings.** The sum of squares, loading of factor extracted in Feelings factor construct, was 85.282%, and factor loadings of all measuring items exceed 0.900.

**Additional File 6: Factor analysis of Clinical outcomes with number of components and total variance explained**

| **Component** | **Initial eigenvalues** | | | **Extraction sums of squared loadings** | | |
| --- | --- | --- | --- | --- | --- | --- |
|  | **Total** | **% of Variance** | **Cumulative %** | **Total** | **% of Variance** | **Cumulative %** |
| 1 | 4.186 | 83.728 | 83.728 | 4.186 | 83.728 | 83.728 |
| 2 | 0.292 | 5.836 | 89.564 | .. | .. | .. |
| 3 | 0.209 | 4.173 | 93.736 | .. | .. | .. |
| 4 | 0.168 | 3.351 | 97.088 | .. | .. | .. |
| 5 | 0.146 | 2.912 | 100.000 | .. | .. | .. |

**Additional File 7: Component matrix of Clinical outcomes**

| **Item** | **Component 1** |
| --- | --- |
| 11 | 0.899 |
| 12 | 0.900 |
| 13 | 0.931 |
| 14 | 0.928 |
| 15 | 0.918 |

**Additional File 6: Factor analysis of Clinical outcomes with number of components and total variance explained and Additional File 7: Component matrix of Clinical outcomes.** The sum of squares, loading of factor extracted in Clinical outcomes factor construct, was 84.728%, and factor loadings of all measuring items were 0.800 above.

**Additional File 8: Factor analysis of Activities with number of components and total variance explained**

| **Component** | **Initial eigenvalues** | | | **Extraction sums of squared loadings** | | |
| --- | --- | --- | --- | --- | --- | --- |
|  | **Total** | **% of Variance** | **Cumulative %** | **Total** | **% of Variance** | **Cumulative %** |
| 1 | 4.332 | 86.634 | 86.634 | 4.332 | 86.634 | 86.634 |
| 2 | 0.215 | 4.301 | 90.935 | .. | .. | .. |
| 3 | 0.175 | 3.507 | 94.442 | .. | .. | .. |
| 4 | 0.154 | 3.086 | 97.528 | .. | .. | .. |
| 5 | 0.124 | 2.472 | 100.000 | .. | .. | .. |

**Additional File 9: Component matrix of Activities**

| **Item** | **Component 1** |
| --- | --- |
| 16 | 0.933 |
| 17 | 0.917 |
| 18 | 0.928 |
| 19 | 0.934 |
| 20 | 0.941 |

**Additional File 8: Factor analysis of Activities with number of components and total variance explained and Additional File 9: Component matrix of Activities.** The sum of squares, loading of factor extracted in Activities factor construct, was 86.634%, and the factor load of each item is more than 0.900.

**Additional File 10: Item-Total Statistics for Pain**

| **Item** | **Scale mean after** **item deleted** | **Scale variance after item deleted** | **Corrected item-total correlation** | **Cronbach’s α coefficient after** **item deleted** |
| --- | --- | --- | --- | --- |
| 1 | 25.51 | 51.269 | 0.856 | 0.937 |
| 2 | 26.24 | 48.752 | 0.870 | 0.935 |
| 3 | 25.19 | 53.619 | 0.804 | 0.946 |
| 4 | 26.03 | 48.816 | 0.911 | 0.927 |
| 5 | 26.07 | 50.133 | 0.857 | 0.937 |

**Additional File 10: Item-Total Statistics for Pain.** The Cronbach’s α coefficient of the Pain dimension was 0.949, and all items in Pain showed a Cronbach’s alpha coefficient over 0.90.

**Additional File 11: Item-Total Statistics for Feelings**

| **Item** | **Scale mean after item deleted** | **Scale variance after item deleted** | **Corrected item-total correlation** | **Cronbach’s α coefficient after item deleted** |
| --- | --- | --- | --- | --- |
| 6 | 27.61 | 51.614 | 0.883 | 0.946 |
| 7 | 27.50 | 52.575 | 0.883 | 0.946 |
| 8 | 27.44 | 52.606 | 0.892 | 0.944 |
| 9 | 27.56 | 53.262 | 0.853 | 0.951 |
| 10 | 27.61 | 52.041 | 0.884 | 0.946 |

**Additional File 11: Item-Total Statistics for Feelings.** The Cronbach’s α coefficient of the Feelings dimension was 0.957, and all items in this dimension showed a Cronbach’s alpha coefficient over 0.90.

**Additional File 12: Item-Total Statistics for Clinical outcomes**

| **Item** | **Scale mean after item deleted** | **Scale variance after item deleted** | **Corrected item-total correlation** | **Cronbach’s α coefficient after item deleted** |
| --- | --- | --- | --- | --- |
| 11 | 26.64 | 56.137 | 0.841 | 0.944 |
| 12 | 26.57 | 56.715 | 0.843 | 0.944 |
| 13 | 26.81 | 54.528 | 0.889 | 0.936 |
| 12 | 26.86 | 53.630 | 0.884 | 0.937 |
| 15 | 27.04 | 53.978 | 0.869 | 0.939 |

**Additional File 12: Item-Total Statistics for Clinical outcomes.** The Cronbach’s α coefficient of the Clinical outcomes dimension was 0.951, and all items in this dimension showed a Cronbach’s alpha coefficient over 0.90.

**Additional File 13: Item-Total Statistics for Activities**

| **Item** | **Scale mean after item deleted** | **Scale variance after item deleted** | **Corrected item-total correlation** | **Cronbach’s α coefficient after item deleted** |
| --- | --- | --- | --- | --- |
| 16 | 25.22 | 64.157 | 0.895 | 0.952 |
| 17 | 25.23 | 65.764 | 0.870 | 0.956 |
| 18 | 25.28 | 64.974 | 0.887 | 0.953 |
| 19 | 25.35 | 64.123 | 0.896 | 0.951 |
| 20 | 25.51 | 63.155 | 0.906 | 0.950 |

**Additional File 13: Item-Total Statistics for Activities.** The Cronbach’s α coefficients of GPS was 0.984, and the Cronbach’s α coefficients of pain, feelings, clinical outcomes, and activities were 0.949, 0.957, 0.951, and 0.961, which demonstrated the scale had excellent reliability.

**Additional File 14:** **The discrimination and difficulty level of the Chinese GPS**

| **Item** | **Discrimination** | **Difficulty level** | | | | | | | | | |
| --- | --- | --- | --- | --- | --- | --- | --- | --- | --- | --- | --- |
| 1 | 2.776 | -1.795 | -1.564 | -1.234 | -0.809 | -0.068 | 0.586 | 1.28 | 1.971 | 2.873 | 3.643 |
| 2 | 2.271 | -1.741 | -1.32 | -0.972 | -0.291 | 0.984 | 1.598 | 2.367 | 2.731 | 3.448 | 4.513 |
| 3 | 2.358 | -2.073 | -1.956 | -1.539 | -1.201 | -0.485 | 0.319 | 1.035 | 1.835 | 2.577 | 3.705 |
| 4 | 2.949 | -1.304 | -0.993 | -0.451 | 0.397 | 1.073 | 1.651 | 2.013 | 2.586 | 3.653 | .. |
| 5 | 2.671 | -1.515 | -1.258 | -0.362 | 0.39 | 1.072 | 1.705 | 2.044 | 2.65 | 3.798 | .. |
| 6 | 3.07 | -1.5 | -1.288 | -1.074 | -0.655 | -0.128 | 0.472 | 1.214 | 1.837 | 2.604 | 3.419 |
| 7 | 2.89 | -1.61 | -1.43 | -1.285 | -0.818 | -0.171 | 0.454 | 1.019 | 1.659 | 2.655 | 3.619 |
| 8 | 3.078 | -1.55 | -1.458 | -1.154 | -0.93 | -0.217 | 0.381 | 1.097 | 1.667 | 2.69 | 3.686 |
| 9 | 2.648 | -1.805 | -1.449 | -1.209 | -0.816 | -0.159 | 0.534 | 1.117 | 1.751 | 2.705 | 3.676 |
| 10 | 2.987 | -1.628 | -1.374 | -1.13 | -0.747 | -0.089 | 0.548 | 1.131 | 1.743 | 2.461 | 3.469 |
| 11 | 2.852 | -1.668 | -1.337 | -1.104 | -0.662 | -0.086 | 0.62 | 1.218 | 1.832 | 2.772 | 3.772 |
| 12 | 2.715 | -1.755 | -1.463 | -1.22 | -0.819 | -0.131 | 0.527 | 1.206 | 1.762 | 2.719 | 3.537 |
| 13 | 3.086 | -1.492 | -1.199 | -1.029 | -0.498 | 0.137 | 0.835 | 1.462 | 2.027 | 2.797 | 4.002 |
| 14 | 3.312 | -1.447 | -1.308 | -1.135 | -0.556 | 0.078 | 0.664 | 1.177 | 1.586 | 2.384 | 3.242 |
| 15 | 2.981 | -1.522 | -1.387 | -1.138 | -0.302 | 0.342 | 0.93 | 1.543 | 2.014 | 2.754 | 3.628 |
| 16 | 3.046 | -1.425 | -1.214 | -0.985 | -0.338 | 0.272 | 0.807 | 1.357 | 1.81 | 2.511 | 3.54 |
| 17 | 2.722 | -1.543 | -1.406 | -1.165 | -0.409 | 0.247 | 0.933 | 1.512 | 1.976 | 2.699 | 3.733 |
| 18 | 2.708 | -1.552 | -1.342 | -1.168 | -0.395 | 0.284 | 0.906 | 1.438 | 1.908 | 2.66 | 3.544 |
| 19 | 2.833 | -1.503 | -1.364 | -1.122 | -0.305 | 0.271 | 0.981 | 1.386 | 1.837 | 2.654 | 3.467 |
| 20 | 2.732 | -1.505 | -1.34 | -1.115 | 0.033 | 0.675 | 1.194 | 1.692 | 2.116 | 2.838 | 3.795 |

**Additional File 14: The discrimination and difficulty level of the Chinese GPS.**
